# Supplementary material for: The E2F4 transcriptional repressor is a key mechanistic regulator of colon cancer resistance to irinotecan (CPT-11)
Source: bioRxiv. 2025 Jan 24:2025.01.22.633435. Preprint. [Version 1] doi: 10.1101/2025.01.22.633435 (PMC11785039; doi:10.1101/2025.01.22.633435)
Supplement: 2 [file NIHPP2025.01.22.633435v1-supplement-2.pdf]

bioRxiv preprint doi: https://doi.org/10.1101/2025.01.22.633435; this version posted January 24, 2025. The copyright holder for this preprint (which was not certified by peer review) is the author/funder, who has granted bioRxiv a license to display the preprint in perpetuity. It is made available under aCC-BY 4.0 International license.

Supplementary Figure S1

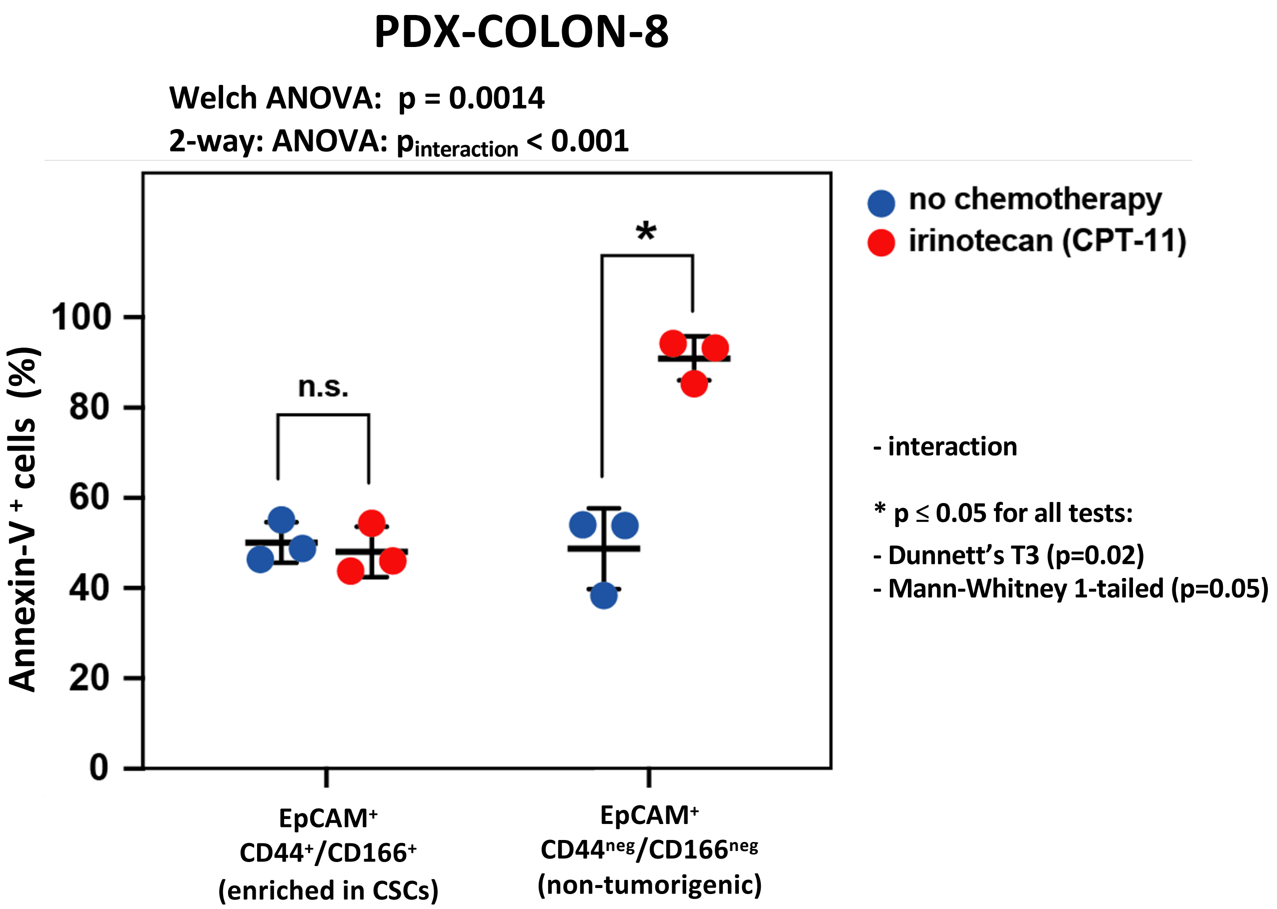

Supplementary Figure S1. Irinotecan (CPT-11) displays preferential cytotoxicity against the subset of colon cancer cells with a *top-of-the-crypt* phenotype (EpCAM$^+$, CD44^{neg}, CD166^{neg}) as compared to the subset with a *bottom-of-the-crypt* phenotype (EpCAM$^+$, CD44$^+$, CD166$^+$). To understand whether colon cancer cells with a *cancer stem cell* (CSC) phenotype displayed preferential resistance to chemotherapy, we tested whether, in immune-deficient mice engrafted with a human colon cancer *patient-derived xenograft* (PDX) line, *in vivo* treatment with irinotecan (CPT-11) was more capable of inducing apoptosis in cancer cells with a *top-of-the-crypt* (EpCAM$^+$, CD44^{neg}, CD166^{neg}) phenotype, which are non-tumorigenic, as compared to cancer cells with a *bottom-of-the-crypt* phenotype (EpCAM$^+$, CD44$^+$, CD166$^+$), which are enriched in *cancer stem cells* (CSCs). Adult, female, NOD/SCID/IL2Rg$^{-/-}$ (NSG) immune-deficient mice were engrafted sub-cutaneously (s.c.) with the PDX-COLON-8 line, which is known to contain populations with both *top-of-the-crypt* (EpCAM$^+$, CD44^{neg}, CD166^{neg}) and *bottom-of-the-crypt* (EpCAM$^+$, CD44$^+$, CD166$^+$) phenotypes. Tumor-bearing mice were then treated with either irinotecan (50 µg/g, once weekly x 4 weeks, i.p.) or a placebo control (1 ml of saline solution, once weekly x 4 weeks, i.p.) and sub-cutaneous tumors harvested 48 hours after the last treatment (day 24). The percentage of apoptotic cells was quantified by flow cytometry, by measuring the percentage of Annexin-V$^+$ cancer cells in each of the two phenotypic sub-populations. *In vivo* treatment with irinotecan (CPT-11) induced preferential apoptosis among cancer cells with a *top-of-the-crypt* phenotype (EpCAM$^+$, CD44^{neg}, CD166^{neg}) as compared to cancer cells with a *bottom-of-the-crypt* phenotype (EpCAM$^+$, CD44$^+$, CD166$^+$). Differences in the percentage of Annexin-V$^+$ cancer cells between populations were tested for statistical significance using; 1) a Welch ANOVA across the full dataset (assuming unequal variance), followed by a Dunnett's T3 test for pre-specified pairwise comparisons; and 2) a two-way ANOVA test for interaction (cell phenotype vs. chemotherapy). Increases in the percentage of Annexin-V$^+$ cancer cells caused by treatment with irinotecan were tested for statistical significance using a one-tailed Mann-Whitney U-test. Error bars: mean +/- standard deviation.

Junichi Matsubara, Yong Fuga Li et al.,

The E2F4 transcriptional repressor is a key mechanistic regulator of colon cancer resistance to irinotecan (CPT-11).

bioRxiv preprint doi: https://doi.org/10.1101/2025.01.22.633435; this version posted January 24, 2025. The copyright holder for this preprint (which was not certified by peer review) is the author/funder, who has granted bioRxiv a license to display the preprint in perpetuity. It is made available under aCC-BY 4.0 International license.

Supplementary Figure S2

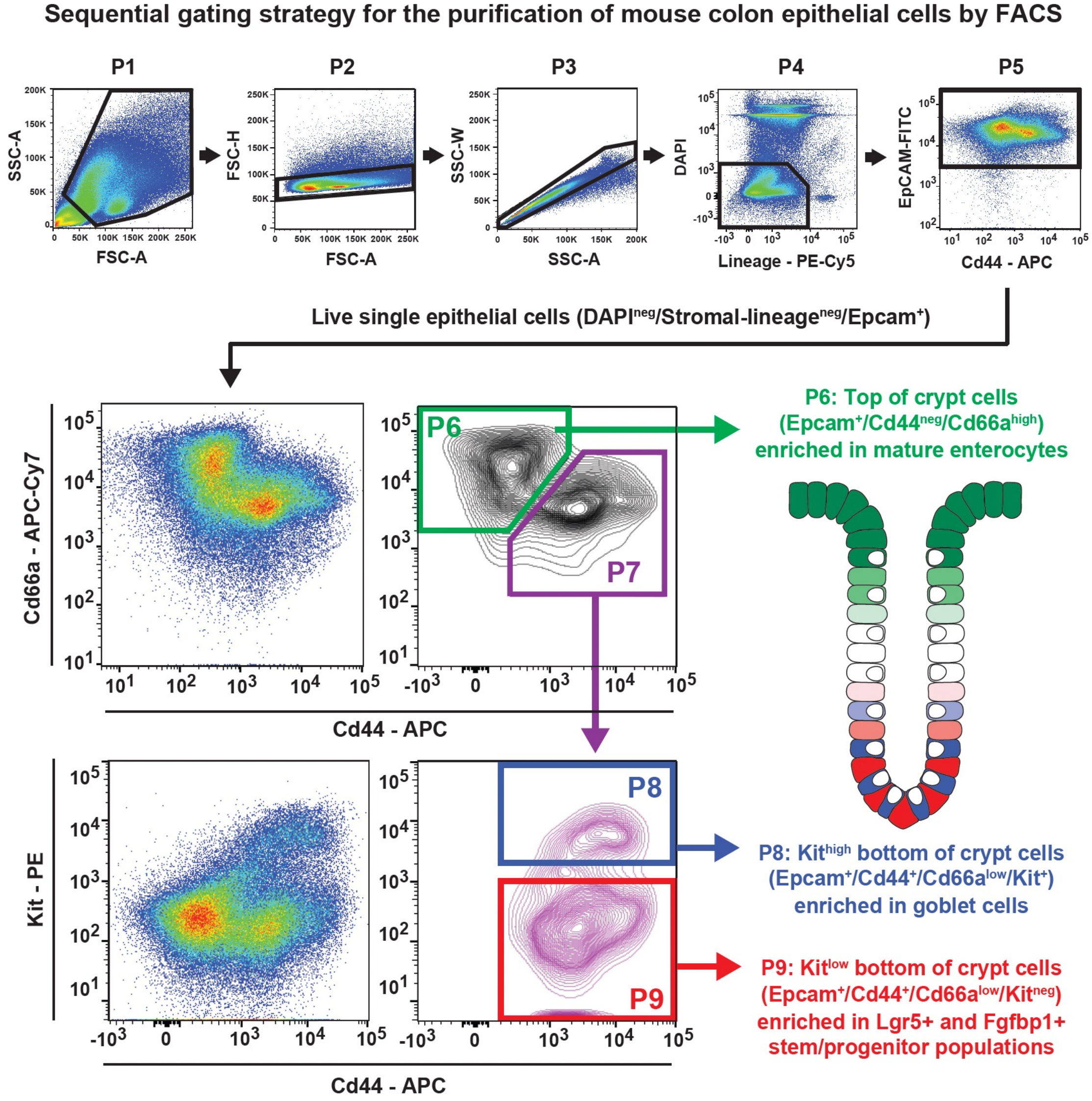

Supplementary Figure S2. Schematic representation of the gating strategy used for the differential purification of distinct sub-types of colon epithelial cells by *fluorescence-activated cell sorting* (FACS). Single-cell suspensions obtained from the dissociation of mouse colonic crypts were sorted by FACS, using nine sequential gates organized hierarchically, and structured as follows: **(P1)** separation of whole cells from small-sized cellular fragments, based on *forward-scatter area* (FSC-A) and *side-scatter area* (SSC-A) profiles; **(P2)** first separation of single-cells from cell-doublets, based on *forward-scatter area* (FSC-A) vs. *forward-scatter height* (FSC-H) profiles; **(P3)** second separation of single-cells from cell-doublets, based on and *side-scatter area* (SSC-A) vs. *side-scatter width* (SSC-W) profiles; **(P4)** separation of live cells of non-stromal lineage ($DAPI^{neg}$/$Lineage^{neg}$) from dead cells ($DAPI^{+}$) and cells of stromal lineage ($Lineage^{+}$); **(P5)** separation of epithelial cells ($EpCAM^{+}$) from non-epithelial cells ($EpCAM^{neg}$); **(P6-P7)** separation of epithelial cells with a *"top-of-the-crypt"* phenotype, which are enriched in enterocytes (P6: $Epcam^{+}$, $CD44^{neg}$, $CD66a^{high}$), from epithelial cells with a *"bottom-of-the-crypt"* phenotype (P7: $Epcam^{+}$, $CD44^{+}$, $CD66a^{low}$); **(P8-P9)** splitting of epithelial cells with a *"bottom-of-the-crypt"* phenotype into a Kit^{+} sub-group (P8), enriched in goblet cells, and a Kit^{neg} sub-group (P9), enriched in both $Lgr5^{+}$ and $Fgfbp1^{+}$ stem/progenitor cells. Cells belonging to stromal lineages ($Lineage^{+}$) were excluded by staining with a cocktail of antibodies directed against surface markers characteristic of hematopoietic and endothelial lineages (Cd3, Cd45, Cd16, Cd31, Cd32).

Junichi Matsubara, Yong Fuga Li et al.,
The E2F4 transcriptional repressor is a key mechanistic regulator of colon cancer resistance to irinotecan (CPT-11).

bioRxiv preprint doi: https://doi.org/10.1101/2025.01.22.633435; this version posted January 24, 2025. The copyright holder for this preprint (which was not certified by peer review) is the author/funder, who has granted bioRxiv a license to display the preprint in perpetuity. It is made available under aCC-BY 4.0 International license.

Supplementary Figure S3

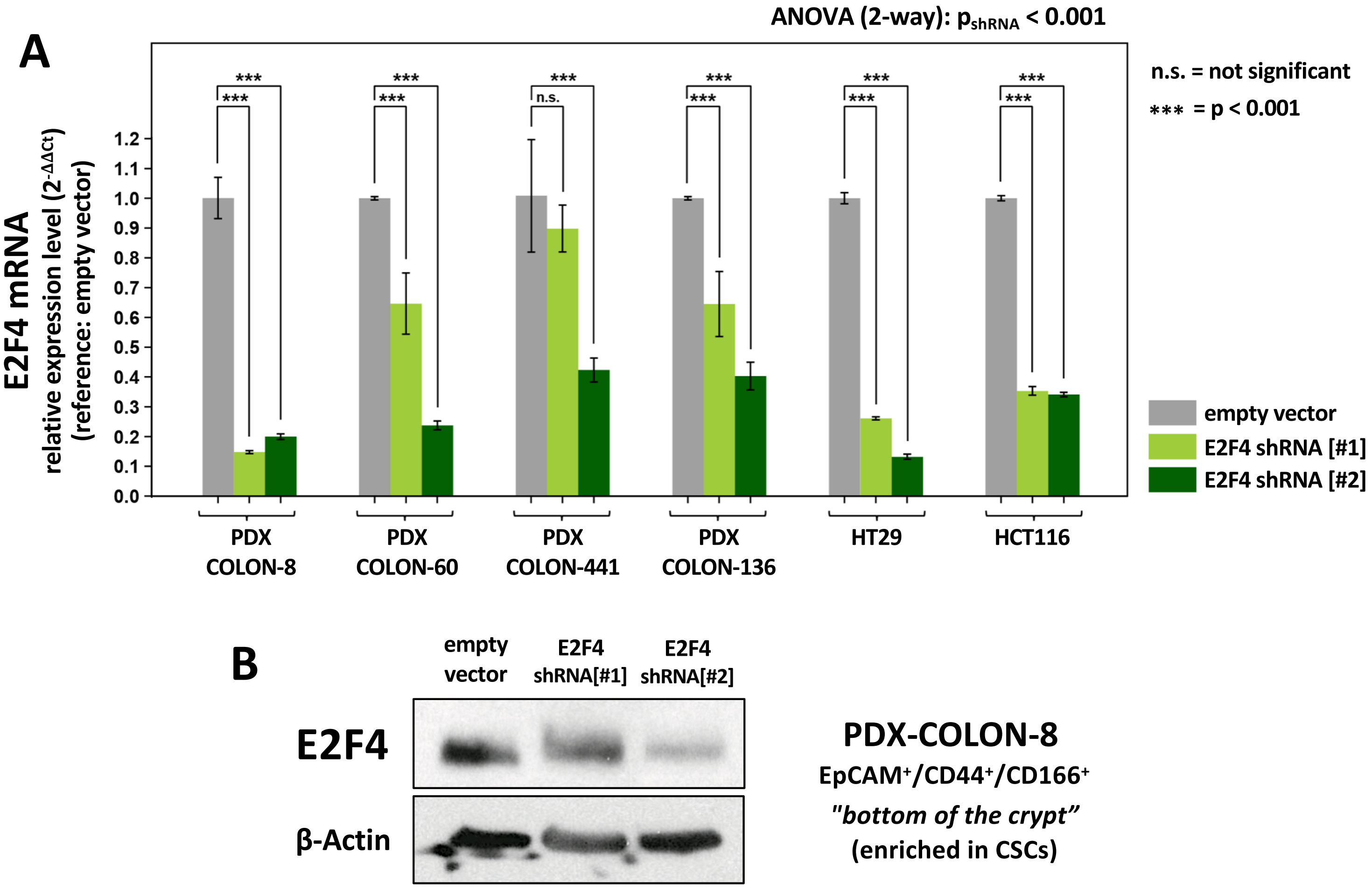

Supplementary Figure S3. Evaluation of E2F4 knock-down efficiency using *short-hairpin RNA* (shRNA) constructs. (A) Analysis by RT-qPCR of *E2F4* mRNA expression in *colorectal cancer* (CRC) cells infected with lentivirus vectors encoding for shRNA constructs targeting the *E2F4* mRNA. The analysis confirmed the capacity of both shRNA constructs used in this study, E2F4-shRNA[#1] and E2F4-shRNA[#2], to cause a reduction in *E2F4* expression levels, as compared to what observed in parental cells infected with an empty vector (reference standard). The analysis was conducted on 6 independent models, including: a) four *patient derived xenograft* (PDX) lines (PDX-COLON-8, PDX-COLON-60, PDX-COLON-C441, PDX-COLON-136); and b) two conventional cell lines grown as *two-dimensional* (2D) monolayers (HT29, HCT116). In both cases, the analysis was conducted on purified preparations of lentivirus-infected malignant cells, isolated by FACS from primary tissues based on the expression of a green fluorescent reporter (copGFP) encoded by the lentivirus construct. Results are reported as fold-changes relative to cells infected with an empty vector, after ΔΔCt normalization to *ACTB* mRNA expression levels and calculation of $2^{-\Delta\Delta Ct}$ values (Livak & Schmittgen, *Methods,* 25:402-408, 2001). Differences in expression levels were tested for statistical significance using a two-way ANOVA test on ΔΔCt values (cell line vs. lentivirus construct) followed by Dunnett's test on pairwise comparisons. Error bars: mean +/- standard deviation. **(B)** Analysis by Western blot of E2F4 protein expression in cancer cells with a *bottom-of-the-crypt* phenotype ($EpCAM^+$, $CD44^+$, $CD166^+$) purified by FACS from the PDX-COLON-8 line following infection with either an empty lentivirus vector or lentivirus vectors encoding for the E2F4-shRNA [#1] or E2F4-shRNA [#2] constructs. The analysis confirmed the capacity of both shRNAs to cause a visually detectable reduction in E2F4 protein levels in the population with a *bottom-of-the-crypt* phenotype ($EpCAM^+$, $CD44^+$, $CD166^+$), which is enriched in cells with *cancer stem cell* (CSC) properties. ACTB (β-Actin) protein levels were analyzed in parallel and used as a visual standard to normalize for the total amount of protein loaded onto the gel.

bioRxiv preprint doi: https://doi.org/10.1101/2025.01.22.633435; this version posted January 24, 2025. The copyright holder for this preprint (which was not certified by peer review) is the author/funder, who has granted bioRxiv a license to display the preprint in perpetuity. It is made available under aCC-BY 4.0 International license.

Supplementary Figure S4

A

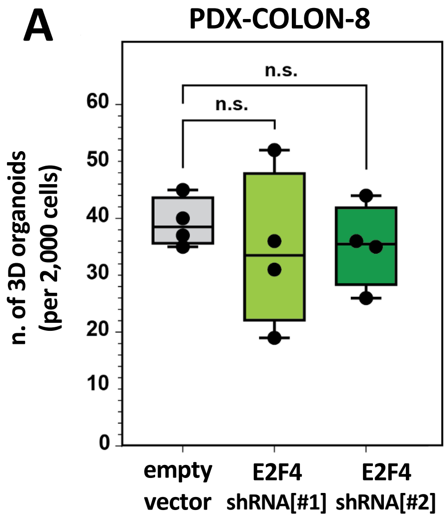

n.s. = not significant

Welch ANOVA + Dunnett's T3 test for pairwise comparisons

B **PDX-COLON-8 - Extreme limiting dilution analysis (ELDA)[1,2]**

Lentivirus construct	n. of tumors/n. of injected mice: 3,000 cells/mouse	500	100	cancer stem cell frequency (95% CI)[3]	p-value (E2F4 vs empty)
**Empty vector**	6/8	4/8	0/12	1/1,658 (1/3,329 - 1/826)	-
**shRNA E2F4 [#2]**	4/4	3/4	2/8	1/355 (1/886 - 1/142)	0.0111

C

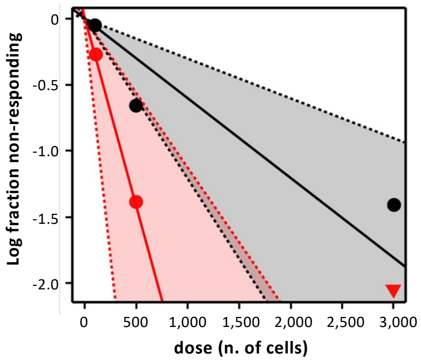

D **PDX-COLON-60 - Extreme limiting dilution analysis (ELDA)[1,2]**

Lentivirus construct	n. of tumors/n. of injected mice: 5,000 cells/mouse	500	100	50	cancer stem cell frequency (95% CI)[3]	p-value (E2F4 vs empty)
**Empty vector**	2/2	2/2	4/4	3/4	1/31 (1/78 - 1/12)	-
**E2F4 shRNA [#2]**	2/2	2/2	4/4	3/4	1/31 (1/78 - 1/12)	N.S.[4]

E

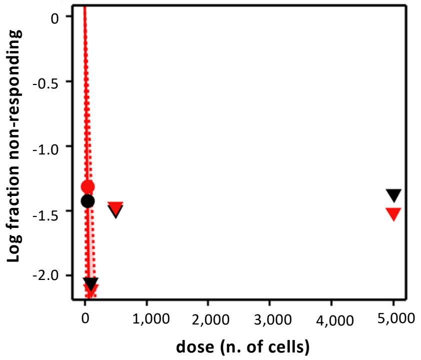

[1] **Hu and Smyth, *Journal of Immunological Methods*, 347:70-78 (2009)**
[2] **http://bioinf.wehi.edu.au/software/elda/**
[3] **CI = confidence interval;** [4] **N.S. = not significant**

Supplementary Figure S4. Downregulation of E2F4 expression does not result in reductions of either the *in vitro* organoid-forming capacity or the *in vivo* tumor-forming capacity of human colon cancer cells. The role of E2F4 in regulating the tumor-initiating capacity of colon cancer cells was evaluated in two *patient-derived xenograft* (PDX) lines infected with lentivirus vectors encoding E2F4-specific *short-hairpin RNA* (shRNA) constructs in tandem with a fluorescent reporter (copGFP). Infected cancer cells were sorted based on copGFP expression and tested prospectively for changes in their capacity to form 3D organoids (*in vitro*) or solid tumors (*in vivo*) as compared to cells infected with an empty vector. **(A)** Comparison of the number of 3D organoids formed by PDX-COLON-8 cancer cells infected with different lentivirus vectors, after plating on Matrigel (n=2,000 cells/well). n.s.: not significant (Welch ANOVA + Dunnett's T3 test for pairwise comparisons). **(B-E)** *Extreme limiting dilution analysis* (ELDA) of the *in vivo* tumorigenic capacity of cancer cells from either the PDX-COLON-8 (B-C) or the PDX-COLON-60 (D-E) lines, following infection with lentivirus vectors encoding E2F4-shRNA constructs. The ELDA was based on the approach described by Hu & Smyth (*J. Immunol. Methods*, 347:70-78, 2009). Cancer cells infected with E2F4-shRNA constructs did not display reductions in their capacity to form organoids (*in vitro*) or solid tumors (*in vivo*) as compared to cells infected with an empty vector.

Junichi Matsubara, Yong Fuga Li et al.,

The E2F4 transcriptional repressor is a key mechanistic regulator of colon cancer resistance to irinotecan (CPT-11).

Supplementary Table 1: Signaling pathways and gene-expression programs identified as differentially modulated between colon cancer cells with a *bottom-of-the-crypt* (EpCAM$^+$, CD44$^+$, CD166$^+$) and a *top-of-the-crypt* (EpCAM$^+$, CD44^{neg}, CD166^{neg}) phenotype, following *in vivo* treatment with irinotecan (CPT-11).

**Signaling pathway or gene-expression program[1,2]**		**Fisher exact test[4]**			**Direction of response**	
**Name**	**Source Database[3]**	**p-value**	**q-value[5]**	**OR[6]**	**OR - CD44$^+$ bottom-crypt[7]**	**OR - CD44^{neg} top-crypt[8]**
Mitotic prometaphase	Reactome	3.11 E-10	4.79 E-07	10.3	0.0	18.7
Aurora B pathway	PID	8.92 E-09	6.88 E-06	16.5	0.0	29.7
Mitotic M-M/G1 phases	Reactome	5.06 E-08	2.60 E-05	6.6	0.0	12.1
DNA replication	Reactome	1.71 E-07	6.58 E-05	6.6	0.0	12.1
PLK1 pathway	PID	3.90 E-07	0.000120	16.9	0.0	30.6
Cell cycle mitotic	Reactome	1.48 E-05	0.003807	5.2	0.0	9.8
Cell cycle	Reactome	2.61 E-05	0.005743	5.7	1.4	9.4
Generic transcription pathway	Reactome	0.000170	0.029090	2.4	0.8	3.7
Kinesins	Reactome	0.000267	0.037417	16.6	0.0	29.6
Cyclin A/B1-associated events during G2/M transition	Reactome	0.001380	0.134185	40.5	0.0	72.5
Glucose metabolism	Reactome	0.007153	0.424517	0.0	0.0	0.0
Processing of capped intron containing pre-mRNA	Reactome	0.191815	0.991098	0.8	0.0	1.5
mRNA processing	Reactome	0.290229	0.991098	0.7	0.0	1.3
Respiratory electron transport	Reactome	0.551698	1.000000	1.8	4.2	0.0

[1]As identified using gene-sets from the *Molecular Signatures Database* (MSigDB) v4.0 (Liberzon *et al.*, *Bioinformatics*, 1739-1740, 2011; https://www.gsea-msigdb.org). [2]Signaling pathways were ranked based on the p-value from a Fisher exact test, assessing their enrichment in genes identified as differentially responsive (upregulated or downregulated) by irinotecan between *bottom-of-the-crypt* and *top-of-the-crypt* cells. [3]Reactome: *Reactome knowledgebase* (Joshi-Tope *et al.*, *Nucleic Acids Research*, 33:D428-432, 2005); PID: *Pathway Interaction Database* (Schaefer *et al.*, *Nucleic Acids Research*, 37:D674-679, 2009). [4]The test evaluates whether genes belonging to a specific signaling pathway are enriched in genes identified as differentially responsive to irinotecan (i.e., genes found at the top 5% or bottom 5% of all genes analyzed by microarrays after ranking based on the interaction parameter of our linear model, corresponding to genes displaying the highest difference, in either direction, when comparing their change in expression following treatment with irinotecan in *bottom-of-the-crypt* and *top-of-the-crypt* cells). [5]Computed based on the Benjamini-Hochberg approach to correct for multiple comparisons. [6]Odds ratio for the enrichment in genes differentially responsive to irinotecan. [7]Odds ratio for the enrichment in genes displaying either a higher induction or lower suppression in *bottom-of-the-crypt* (EpCAM$^+$, CD44$^+$, CD166$^+$) cells (i.e., top 5% after ranking based on interaction parameter). [8]Odds ratio for the enrichment in genes displaying either a higher induction or lower suppression in *top-of-the-crypt* (EpCAM$^+$, CD44^{neg}, CD166^{neg}) cells (i.e., bottom 5% after ranking based on interaction parameter).

bioRxiv preprint doi: https://doi.org/10.1101/2025.01.22.633435; this version posted January 24, 2025. The copyright holder for this preprint (which was not certified by peer review) is the author/funder, who has granted bioRxiv a license to display the preprint in perpetuity. It is made available under aCC-BY 4.0 International license.

Supplementary Table 3: Clinical and pathological characteristics of the human *colorectal carcinomas* (CRCs) from which the *patient derived xenograft* (PDX) models utilized in this study were established.

PDX line	Patient		primary site of origin	primary vs. metastasis	Tumor stage[1]		Tumor grade[2]
	age	sex			TNM	AJCC	
PDX-COLON-8	49	male	Sigmoid colon	primary	T3N0	IIa	G2
PDX-COLON-60	58	male	Right colon	primary	T4aN2bM1b	IVb	G2
PDX-COLON-136	74	female	Right colon	primary	T4N1bM0	IIIb	G3
PDX-COLON-441	n.a.	male	Left colon	primary	T3N2bM0	IIIc	n.a.

[1] *Tumor-Node-Metastasis* (TNM) status and pathological stage are reported according to the 7th edition of the *American Joint Committee on Cancer* (AJCC) staging manual for colorectal cancer (2009). [2] Grade of histological differentiation, annotated as follows: G1, well differentiated; G2: moderately differentiated; G3, poorly differentiated; n.a., not available.

bioRxiv preprint doi: https://doi.org/10.1101/2025.01.22.633435; this version posted January 24, 2025. The copyright holder for this preprint (which was not certified by peer review) is the author/funder, who has granted bioRxiv a license to display the preprint in perpetuity. It is made available under aCC-BY 4.0 International license.

Supplementary Table 4: Molecular properties of the *short hairpin RNA* (shRNA) constructs used to knock-down E2F4 protein expression in this study.

shRNA	Construct ID[1,2]	mRNA target site[3]	off-target genes[4]	sequence of shRNA strands
E2F4 shRNA [#1]	TRCN0000013808	nt. 1933<br>3'UTR	none detected	sense: 5'-CCCTCTCTTCATTTCGGCTTT-3'<br>antisense: 5'-AAAGCCGAAATGAAGAGAGGG-3'
E2F4 shRNA [#2]	TRCN0000013809	nt. 242<br>CDS	none detected	sense: 5'-CCCTCTCTTCATTTCGGCTTT-3'<br>antisense: 5'-AAAGCCGAAATGAAGAGAGGG-3'

[1] The two shRNA constructs were developed by *The RNAi Consortium* (TRC) at the *Broad Institute* and are available from *Horizon* (Perkin Elmer). [2] The two shRNA constructs were subcloned into the *pSIH1-H1-copGFP* lentivirus vector (*System Biosciences*), which expresses the *copepod green fluorescent protein* (copGFP) as a fluorescent reporter. [3] Target sites in the *E2F4* mRNA are reported as the first nucleotide of the target sequence within the NM_001950.4 transcript from the NCBI RefSeq database (https://www.ncbi.nlm.nih.gov/refseq); 3'UTR: 3' untranslated region; CDS: coding sequence. [4]As reported by the current analysis by the *Gene Perturbation Portal* (GPP), managed by the *Broad Institute* (https://portals.broadinstitute.org/gpp/public).
